# Mutations of Novel Influenza A(H10N8) Virus in Chicken Eggs and MDCK Cells

**DOI:** 10.3201/eid2009.140257

**Published:** 2014-09

**Authors:** Jian Yang, Ting Zhang, Li Guo, Yongfeng Hu, Jinlin Li, Haoxiang Su, Yan Xiao, Xianwen Ren, Jie Dong, Lilian Sun, Yan Xiao, Li Li, Fan Yang, Jianwei Wang, Hui Yuan, Qi Jin

**Affiliations:** MOH Key Laboratory of Systems Biology of Pathogens, Institute of Pathogen Biology, Chinese Academy of Medical Sciences and Peking Union Medical College, Beijing, China (J. Yang, T. Zhang, L. Guo, Y. Hu, J. Li, H. Su, Y. Xiao, X. Ren, J. Dong, L. Sun, Y. Xiao, L. Li, F. Yang, J. Wang, Q. Jin);; Jiangxi Provincial Center for Disease Control and Prevention, Nanchang, China (H. Yuan)

**Keywords:** influenza A virus, mutation, genome, avian-origin, antiviral drug resistance, viruses, A(H10N8)

## Abstract

The recent emergence of human infection with influenza A(H10N8) virus is an urgent public health concern. Genomic analysis showed that the virus was conserved in chicken eggs but presented substantial adaptive mutations in MDCK cells. Our results provide additional evidence for the avian origin of this influenza virus.

Influenza A virus remains a major threat to public health worldwide. The 2000s witnessed the epidemic of human infections with the avian influenza A(H5N1) and A(H7N9) viruses in China (*1*,*2*) and a global pandemic of human influenza caused by a novel swine-origin influenza A(H1N1) virus (*3*). More recently, the first human case of a novel influenza A(H10N8) virus infection was reported in China, and 2 additional human cases have been confirmed in the same province (*4*,*5*). The emergence of the novel influenza A(H10N8) virus has become an urgent public health concern (*6*).

A preliminary genomic analysis showed that the emerging influenza virus was genetically distinct from the avian influenza A(H10N8) viruses previously identified in China, and scientists have postulated that the virus resulted from multiple reassortments of subtype H9N2 strains that circulated widely in poultry in China (*4*). Nevertheless, no identical influenza A(H10N8) virus was detected in the live-poultry market visited by the first patient before the onset of her illness, and the origin of the novel A(H10N8) virus remains unclear. We compared the genomic mutations of the virus cultured in embryonated chicken eggs and in MDCK cells in an attempt to find additional evidence to support the possible avian origin of the virus.

## The Study

We obtained a tracheal aspirate specimen from the lower respiratory tract of the patient with the first reported A(H10N8) virus infection on day 7 after illness onset (*4*). The tracheal aspirate specimen was inoculated on monolayers of MDCK cells and into the allantoic cavities of 10-day-old embryonated chicken eggs. The isolated novel A(H10N8) virus strain IPB13 was serially passaged in MDCK cells every 72 hours for a total of 3 passages. After 1 blind passage, cytopathic effects were clearly visible in ≈80% of the cells. The supernatants of the second and third passages in MDCK cells and the allantoic fluids of the inoculated chicken eggs were individually harvested and processed for full genome sequencing. We extracted total RNA from each sample using a QIAamp Viral RNA Mini Kit (QIAGEN, Hilden, Germany) and amplified it by sequence-independent PCR with a OneStep RT-PCR Kit (QIAGEN). We determined the viral genome sequence of each isolate using an ABI 3730xl automatic DNA analyzer (Life Technologies, Grand Island, NY, USA). Potential heterogeneous sites in each genome were initially identified by manual inspection of the sequence trace data and confirmed by clone sequencing as previously described (*7*).

In addition, the tracheal aspirate sample was processed for direct deep sequencing as previously described (*8*) but with a slight modification. We used an Illumina/HiSeq2500 sequencer (Illumina, San Diego, CA, USA) to generate 100-bp single-end reads according to the manufacturer’s instructions. The deep sequencing reads were screened for quality control and removal of human contamination as previously described (*9*). The valid reads were aligned to the previously determined genome sequence of the novel A(H10N8) virus strain JX346 (GISAID [Global Initiative on Sharing All Influenza Data] accession no. EPI497477–84) by using the BWA program with default parameters (*10*). We obtained a total of 2,629,199 reads that were successfully aligned to the JX346 genome. Then, we used the SAMtools package to detect possible polymorphic or heterogeneous sites from the alignment (*11*). We deposited the complete genome sequences of all isolates in GenBank under accession nos. KJ406531–KJ406562.

The change of host environment from the in vivo human respiratory tract to the in vitro cell lines might introduce mutations in the virus genome during culture. Indeed, in our previous study, we observed dozens of point mutations in the genome of novel A(H7N9) virus obtained from embryonated chicken eggs compared with that derived directly from clinical sample (*8*). However, in this study, we obtained exactly the same genome sequences of the novel A(H10N8) virus from the clinical sample and that cultured in chicken eggs (Table). Moreover, the 2 heterogeneous sites in the clinical sample were retained during culture in embryonated chicken eggs. In contrast, 10 heterogeneous sites, 8 of which resulted in point mutations, were observed in the virus genomes derived from cultures in MDCK cells (Table). Additionally, no genomic difference was visible between viruses in the second and third passages in MDCK cells.

**Table:** Mutations and heterogeneities of influenza A(H10N8) virus detected in a tracheal aspirate sample from a human, the first passage in embryonated chicken eggs, and the second and third passages in MDCK cells

Segment	Nucleotide position†	Direct sequencing	Embryonated chicken eggs P1	MDCK cells		Amino acid change‡
				P2	P3	
PB2	1,879	A→G	A→G	A	A	E627K§
PA	1,689	G	G	A→G	A→G	–
PA	1,995	G	G	A→G	A→G	–
PA	1,997	T	T	C→T	C→T	L666P
HA	688	A	A	G→A	G→A	R220G
NP	583	C	C	A→C	A→C	–
NP	1,405	G	G	A→G	A→G	E469K
NA	739	C	C	C→T	C→T	A246V§
NA	877	G	G	A→G	A→G	R292K
NA	1,187	C	C	C→T	C→T	–
NS1	641	G→A	G→A	A→G	A→G	E208K

*HA, hemagglutinin; NA, neuraminidase; NP, nucleocapsid protein; NS, nonstructural protein; PA, polymerase acidic; PB, polymerase basic; –, synonymous substitution without amino acid change. †Numbered from the first nucleotide of the determined genomic sequence. ‡Site positions are numbered from the start codon (M); H3 and N2 numbering for HA and NA, respectively. §Potential amino acid change caused by the minor nucleotide in the heterogeneous site.

The heterogeneous site in polymerase basic (PB) 2 resulted in a mixture of glutamic acid and lysine at residue 627 of the PB2 protein in the clinical sample (Table); this mixture might have partially contributed to the severity of the patient’s illness (*4*). Indeed, the heterogeneity in this site vanished during culture in MDCK cells, leading to complete lysine substitution at residue 627 of the PB2 protein as previously observed (*12*). This finding is consistent with the established hypothesis that the E627K substitution in PB2 was associated with the increased transmissibility and pathogenicity of avian influenza viruses in mammals (*13*,*14*). Notably, a heterogeneous site in the neuraminidase (NA) segment led to a mixture of arginine and lysine at residue 292 (N2 numbering) of the NA protein in the viruses cultured in MDCK cells (Table). The R292K substitution in the NA protein of avian influenza A(H7N9) virus was able to reduce the antiviral efficacy of NA inhibitors, especially oseltamivir (*15*). Therefore, although the novel A(H10N8) virus was sensitive to oseltamivir in vitro (*4*), its potential to develop antiviral drug resistance in mammalian cells requires further attention.

## Conclusions

We investigated the genomic mutations and heterogeneities of the novel influenza A(H10N8) virus during culture in embryonated chicken eggs and MDCK cells compared with the genome sequence obtained directly from the clinical specimen. The viral genome was highly conserved during culture in embryonated chicken eggs, and no mutations were identified. This result suggests that the novel A(H10N8) virus might have been highly adapted to an avian-like host before it was transmitted to the human host (i.e., the first patient). In contrast, substantial genetic mutations were observed in the viral genome during culture in MDCK cells; this finding implies an ongoing adaptive microevolution of the virus in a mammalian environment. Taken together, our results favor the proposal that the novel influenza A(H10N8) virus has an avian origin; however, more research is required to establish the definite origin of the emerging influenza virus. Furthermore, the substitutions E627K (in the PB2 protein) and R292K (in the NA protein) observed in the cultures of the MDCK cells indicate that the virus might be undergoing rapid adaptation to mammals and developing antiviral drug resistance. Although only 3 human cases of infection with the novel A(H10N8) virus have been reported, the potential for this virus to threaten public health should not be underestimated.
